# Downregulation of Brain Enriched Type 2 MAGEs Is Associated With Immune Infiltration and Poor Prognosis in Glioma

**DOI:** 10.3389/fonc.2020.573378

**Published:** 2020-12-23

**Authors:** Mohit Arora, Sarita Kumari, Jay Singh, Anita Chopra, Shyam S. Chauhan

**Affiliations:** ^1^Department of Biochemistry, All India Institute of Medical Sciences, New Delhi, India; ^2^Laboratory Oncology Unit, Dr. B. R. Ambedkar Institute Rotary Cancer Hospital (Dr. BRA-IRCH), All India Institute of Medical Sciences, New Delhi, India

**Keywords:** glioma, glioblastoma, melanoma associated antigen, The Cancer Genome Atlas (TCGA), epigenetics, DNA methylation

## Abstract

Melanoma associated antigen (MAGE) is an extensively studied family of tumor-associated genes that share a common MAGE homology domain (MHD). Based upon their expression pattern, MAGE genes have been broadly classified into type 1 MAGEs (T1Ms) and type 2 MAGEs (T2Ms) categories. Interestingly, several T2Ms are highly expressed in the brain and involved in the regulation of neuronal development, differentiation, and survival. Available literature suggests possible tumor suppressor functions of a few T2Ms, while information available about their expression, regulation, and clinical significance in glioma is scanty. This prompted us to perform a comprehensive analysis of T2M expression in glioma. Gene expression data from glioma datasets: Oncomine, TCGA, and REMBRANDT study, were used to assess the mRNA expression of T2M genes (*MAGED1, MAGED2, MAGED3, MAGED4, MAGED4B, MAGEE1, MAGEE2, MAGEF1, MAGEH1, MAGEL2, NSMCE3*, and *NDN*), and their association with clinical characteristics and composition of the tumor microenvironment. Further, mutation, copy number alteration, and DNA methylation data from TCGA were assessed for determining potential mechanisms of T2Ms expression in glioma. Expression analysis revealed overexpression of MAGED subfamily genes in glioma, while other genes of this family exhibited reduced expression in advanced grades of this malignancy. Further, the expression of T2Ms exhibited varying extent of positive correlations with each other. Amongst downregulated T2Ms, *MAGEH1* expression exhibited negative correlations with DNA methylation. Additionally, genes associated with *MAGEH1* were enriched in Myc and Hedgehog signaling. Furthermore, T2Ms downregulation was associated with immune infiltration in glioma tissues and poor overall survival of glioma patients. In multivariate Cox regression analysis, *MAGEH1* emerged as an independent prognosticator in lower grade glioma. Conclusively, these results suggest that expression of T2Ms is associated with important clinical and molecular features in glioma. Mechanistic studies may further provide novel insights into their role in glioma progression.

## Introduction

Gliomas are a group of heterogeneous primary malignant brain tumors with a dismal outcome (1). Histopathological classification of glioma has been constantly evolving with the most recent WHO classification, 2016, classifying gliomas as astrocytic, oligodendroglial or mixed oligo-astrocytic, and further graded as WHO grade I and II (low grades), III (anaplastic) or IV (glioblastoma multiforme) (2). Molecular alterations in glioma including genetic as well as epigenetic changes have been constantly determined and integrated into common clinical practice (2, 3). Isocitrate dehydrogenase gene (*IDH*) mutation and co-deletion of chromosome 1p and 19q (1p/19q codeletion) are frequently observed in low grade gliomas and associated with better patient prognosis (4). IDH mutation confers global epigenomic changes, commonly called the CpG island methylation phenotype (G-CIMP) leading to suppression of tumor suppressor genes (5). Also, methylation O-6-methylguanine-DNA methyltransferase (*MGMT*) gene promoter is associated with better response to temozolomide (TMZ) chemotherapy (6). While these biomarkers help in the management of glioma with the current treatment regimen, identification of novel molecular features may provide better therapeutic opportunities in glioma.

Melanoma associated antigen (MAGE) gene family consists of more than 50 genes that share a common *MAGE* homology domain (MHD) (7, 8). MAGE genes evolved through transposition and segmental duplications in the genome (9). Based on chromosomal location and pattern of gene expression, members of this family are further subdivided into two types. Type 1 MAGEs (T1Ms) include MAGE-A, B, and C subfamilies. The human T1Ms are present as clusters on the X chromosome. T1Ms display germ cell and cancer specific expression, and thereby fulfill the criteria of cancer testis antigens. T1M peptides are recognized by cytotoxic T lymphocytes (CTLs) in a variety of cancers and serve as ideal candidate antigens for tumor vaccines (10–12). Human Genome Organisation (HUGO) has approved the inclusion of twelve Type 2 MAGEs (T2Ms), including *MAGED1*, *MAGED2*, *MAGED3* (*TRO*), *MAGED4*, *MAGED4B*, *MAGEE1*, *MAGEE2*, *MAGEF1*, *MAGEH1*, *MAGEL2*, *NSMCE3*, and *NDN* (Necdin). T2Ms are not restricted to X chromosome and are expressed in a variety of tissues.

The current knowledge of the physiological functions of the MAGE genes is mostly related to T1Ms. Several T1Ms form complex with E3 RING ubiquitin ligases to form MAGE-RING ligases (MRLs) that are involved in ubiquitination mediated protein degradation and other cellular processes such as regulation of transcription and cell cycle (8). T1Ms have also been implicated in apoptosis, cancer cell invasion (13), stem cell maintenance, and DNA repair (14). However, information about the functions of T2Ms is limited. *MAGED1*, *MAGEH1*, and *NDN* are highly expressed in the brain and involved in neuronal development (8, 15, 16). *MAGEE1* and *MAGED4* were initially identified as brain specific members of the MAGE gene family and were significantly enriched in glial tissues (17, 18). However, Necdin is a neuron specific growth suppressor, is downregulated in tumors, including glioma and tumor cell lines, thereby confirming its tumor suppressor functions (19, 20). Low frequency mutations of *MAGEH1* in glioma have been shown to affect its nuclear localization (21). Furthermore, aberrant *MAGEH1* expression has been linked to dementia (22). These studies suggest crucial roles of T2Ms in neuronal growth, survival and possibly in the pathogenesis of central nervous system diseases, including glioma.

Although wide variations in the nucleotide sequences of regulatory regions of T1Ms enables their differential expression (9), epigenetic alterations including DNA methylation and histone modification underlie the co-expression of several T1Ms and other tumor associated antigens in cancers (23). On the contrary, all T2Ms display striking sequence homology in their regulatory regions which possibly permit a common mechanism to control their expression (9). Although contradictory reports also exist regarding maternal imprinting of the Necdin gene (24, 25), epigenetic regulation of *MAGED4* in glioma is documented (18). While current evidence suggests crucial roles of T2Ms in normal brain functions and neurological disorders, no systematic study has been done to investigate the expression, regulation, cellular function, and clinical significance of T2Ms in glioma. Therefore, the current study was undertaken to investigate the regulatory mechanism responsible for altered expression of T2Ms, their cellular functions, and clinical significance in glioma using large scale multi-omics datasets.

## Materials and Methods

### Cell Culture

Human glioblastoma cells U87MG and LN229 (National Centre for Cell Science, Pune, India) were grown in a medium containing RPMI-1640 (HiMedia Laboratories, India), supplemented with 10% (v/v) Fetal bovine serum (Gibco Thermo Fisher Scientific Inc.), 1% penicillin and streptomycin (Gibco, Thermo Fisher Scientific Inc.) in a humidified atmosphere containing 5% CO2 at 37°C.

### 5’Azacytidine Treatment and qRT-PCR

Human glioblastoma cells (2X 10^4^) were plated in each well of 6 well dish. The next day the cells were treated with 1 µM Azacytidine (#A2385, Sigma-Aldrich). After 48 h the cells were washed twice with ice-cold PBS and processed for total cellular RNA isolation using Trizol reagent (Thermo Fisher Scientific Inc.) as per manufacturer’s protocol. One µg of total RNA was reverse transcribed using random hexamers, dNTPs, and M-MuLV reverse transcriptase enzyme (Fermentas, USA). The *MAGEH1* expression was determined by quantitative Real-time PCR using primers Forward, 5’-CGGAGCAATTTTCAGGGCAC-3’; Reverse, 5’- AGCACTTTCCAGACCAGAGC-3’. The mRNA expression of *MAGEH1* was normalized to 18S ribosomal RNA using primers: Forward, 5’-GTAACCCGTTGAACCCCATT-3’; Reverse, 5’-CCATCCAATCGGTAGTAGCG-3’. Ct values for each PCR were analyzed by the 2^-ΔCt^ method. Total cellular RNA isolated from vehicle-treated glioblastoma cells was processed identically and served as control. Depicted results were drawn based on three biological replicates.

### Data Retrieval

For mRNA expression analysis, two datasets were utilized. First, RNA seq data of TCGA-LGG-GBM dataset, which was originally sourced from Broad GDAC Firehose (http://gdac.broadinstitute.org/) contained 515 patients (TCGA-LGG dataset, grade 2 and grade 3 combined) and 152 GBM samples (TCGA-GBM dataset). Second, microarray gene expression data from REMBRANDT (REpository for Molecular BRAin Neoplasia DaTa) was sourced from NCBI-GEO (accession number GSE108476, https://www.ncbi.nlm.nih.gov/geo/) consisting 28 non-tumor brain tissues, 225 lower grade (grade II +III), and 219 GBM tumor samples. Both the datasets were accessed from the GlioVis web server (http://gliovis.bioinfo.cnio.es/) and used for analysis using tools provided in the webserver (26–28). Expression, correlation, and survival analysis were performed using default parameters, without modifications.

### Correlation and Pathway Enrichment Analysis

For correlation analysis in TCGA-LGG and GBM data, the correlation module of the GlioVis tool was used. Gene expression data from three randomly chosen TCGA datasets including TCGA skin cutaneous melanoma, TCGA breast cancer and TCGA lung squamous cell carcinoma, along with CCLE pan-cancer cell line data of 1156 cell lines, were downloaded from cBioPortal website (https://www.cbioportal.org/) (29, 30). Spearman’s correlation analysis was performed followed by heatmap generation and hierarchical clustering using HemI software (31). The default parameters of hierarchical clustering using the average linkage method and Pearson distance were used. Similarly, whole transcriptome correlations of *MAGEH1* were extracted for TCGA-LGG dataset using cBioPortal. After applying a filter for a cutoff of FDR corrected p-value of 0.05 for Spearman’s r value, a total of 13,536 genes with Spearman’s r value ranging from 0.689 to -0.660 were available for gene set enrichment analysis in GSEA software (Broad Institute, http://www.broad.mit.edu/gsea/) (32). Predefined molecular signature database hallmark gene set (version 7.1) was used as a reference gene set for pathway enrichment (33).

### DNA Methylation Analysis

DNA methylation of T2Ms in TCGA cancer datasets was estimated and visualized using MEXPRESS web server (https://mexpress.be) (34, 35). The MEXPRESS web server uses DNA methylation data of cancer and normal tissues from TCGA datasets, which were originally developed on the “Illumina 450k Beadchip” platform. The predesignated methylation probes for each gene were taken into consideration.

### Survival Analysis

Kaplan-Meier survival analysis was performed using a survival tool available at the GlioVis web server, which utilized the “survival” package in R to generate Kaplan-Meier plots. Hazard ratios are determined to utilize the “coxph” function from the “survival” package. For Kaplan-Meier analysis, Patients were distributed in high and low expression groups based on optimal cutoff determined using maximally selected rank statistics (maxstat) function for continuous variables, as provided in the “survminer” package. For survival analysis using univariate and multivariate Cox regression, *MAGEH1* and *MAGED1* expression was taken as a continuous variable. For TCGA-LGG and TCGA-GBM datasets, information on overall survival, disease-specific survival, progression-free interval, and the disease-free interval was available. For, TCGA-GBM data, disease-free interval events were excluded as suggested previously (36).

### TIMER Analysis

Tumor immune estimation score (TIMER) database (https://cistrome.shinyapps.io/timer/), which utilized the RNA sequencing data from TCGA for estimating the correlation between gene expression and level of tumor-infiltrating immune cells (37). We utilized TIMER to calculate the association between gene expression of T2Ms with tumor purity and infiltration of immune cells including B cells, CD4+ T cells, CD8+ T cells, neutrophils, macrophages and dendritic cells in LGG and GBM datasets.

### Statistical Analysis

Data analysis was performed using Graphpad (version 6) and Stata software (version 11). Chi-square test was used to calculate the expression fold change with threshold *p*-value <0.001) between normal and glioma in Oncomine datasets analysis. Mann-Whitney U-test was used for comparison among histological subtypes, molecular subtype and grades (**p<0.001; **p<0.01; *p<0.05; ns, p>0.05). Pearson correlation was used to calculate the correlation of DNA methylation of T2M genes to its expression in LGG and GBM dataset. Kaplan-Meier survival analysis was performed using Wilcoxon and log-rank test, *p*-value <0.05 was considered as statistically significant.

## Results

### Expression Pattern of T2Ms in Glioma

**Figure 1:**
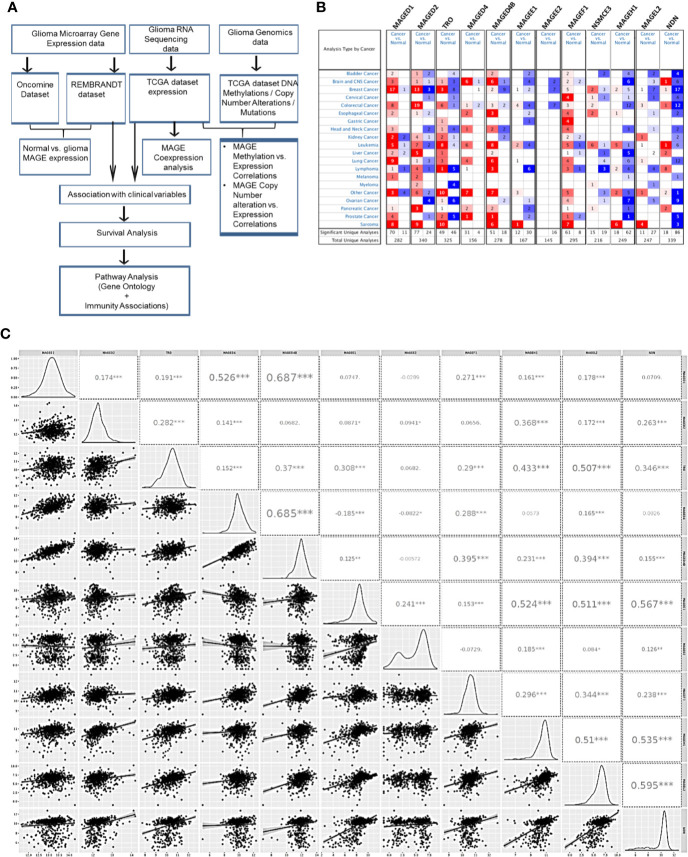
Type 2 MAGE expression in glioma. **(A)** Outline of the current study. **(B)** Oncomine analysis showing the general pattern of overexpression (red color)/downregulation (blue color) of T2Ms in cancer vs. normal tissues in different cancers. **(C)** Correlation matrix of T2Ms in a combined group of TCGA lower grade glioma and glioblastoma dataset. The distribution of variables is shown in left while Pearson correlation coefficients along with the level of significance have been shown on right. (***p<0.001; **p<0.01; *p<0.05).

To compare the expression of T2Ms in glioma with normal brain tissue, we utilized publicly available gene expression datasets: Oncomine, TCGA, and REMBRANDT study. The outline of the study has been depicted in **Figure 1A**. Oncomine analysis was performed to compare gene expression in multiple datasets in parallel to get reliable information regarding the change in T2Ms gene expression (**Figure 1B**). Interestingly, Oncomine analysis revealed that in glioma, several MAGE genes, including *MAGED1*, *MAGED4*, and *MAGED4B* exhibited elevated expression (**Figure 1B**). In contrast, an opposite pattern was observed for *TRO* (MAGED3), *MAGEE1*, *MAGEE2*, *MAGEH1*, *MAGEL2*, and *NDN*, which exhibited reduced expression in tumors compared to normal brain tissues. *NSMCE3*, *MAGED2*, and *MAGEF1* did not exhibit a clear pattern of overexpression/downregulation in Oncomine analysis.

We further analyzed the coexpression pattern of T2Ms in TCGA datasets of glioma and the results are presented in **Figure 1C**. This analysis revealed a strong positive correlation among *MAGED1*, *MAGED4*, and *MAGED4B* (r>0.4) while they exhibited a comparatively weak correlation (r <0.3) to other MAGEs. Similarly, *TRO*, *MAGEE1*, *MAGEH1*, *MAGEL2*, and *NDN* exhibited positive correlations (r >0.3), while others exhibited varying levels of weaker correlations (r <0.3) (**Figure 1C**). We further performed similar co-expression analysis on gene expression datasets for lower grade glioma (LGG) and glioblastoma multiforme (GBM), separately, and observed broadly similar coexpression pattern of T2Ms in LGG and GBM (**Supplementary Figures S1A, B**). Furthermore, we also assessed the correlation of T2Ms expression in other TCGA cancer datasets to determine whether these coexpression patterns are unique to glioma or are common to most cancers. Interestingly, we observed that in TCGA datasets of cutaneous melanoma (TCGA-SKCM), breast cancer (TCGA-BRCA), and lung squamous cell carcinoma (TCGA-LUSC), T1Ms and T2Ms form distinct clusters (**Supplementary Figures S2A–C**). However, within the cluster, they display varying degrees of positive correlations, thereby supporting the possibility of common regulatory mechanisms for their expression. These co-expression patterns also reflected well in a pan-cancer cell line gene expression dataset from Cancer Cell Line Encyclopedia (CCLE, **Supplementary Figure S2D**). Therefore, expression of T2Ms is highly coordinated in two distinct subgroups of T2Ms in glioma, one of which is overexpressed while the other exhibits downregulation in glioma. In view of the reported overexpression of *MAGED4* in glioma (18), we focused our further analysis on downregulated T2Ms using *MAGEH1* as a representative member of this highly co-expressed T2M subgroup. Interestingly, all T2Ms which were found to be downregulated in the expression analysis exhibited preferential enrichment in the brain, as determined by the Genotype-Tissue Expression (GTEx) project whole-body gene expression data (**Supplementary Figures S3A–E**).

### Expression of T2Ms in Different Histological Subtypes

**Figure 2:**
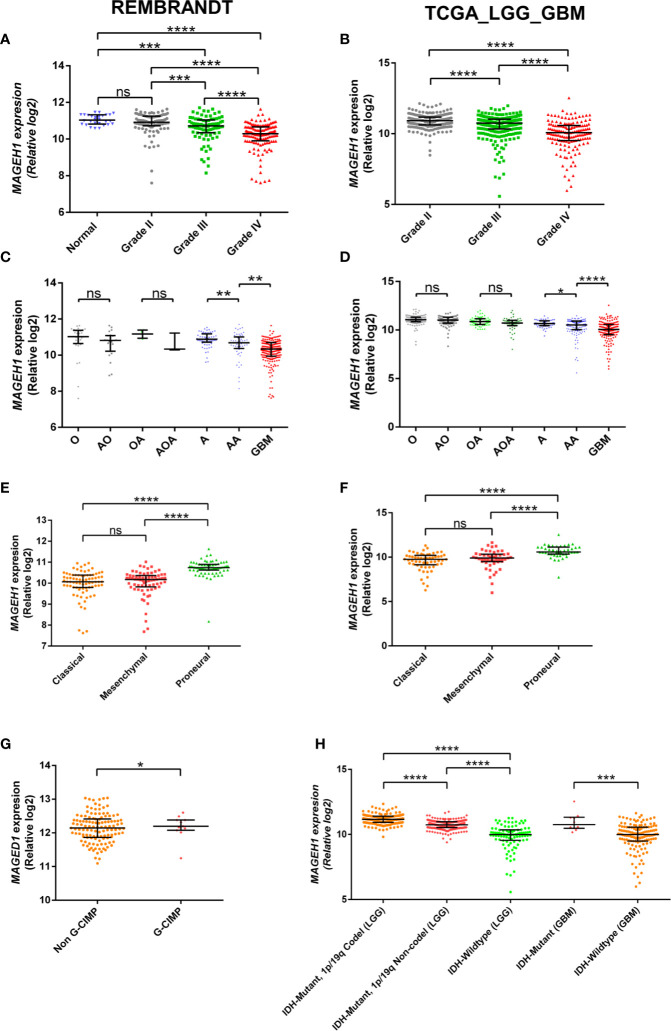
*MAGEH1* gene expression in glioma datasets, Rembrandt (left) and TCGA-LGG-GBM dataset (right) for **(A, B)** Different glioma grades, **(C, D)** Histological subtypes of LGG with GBM, **(E, F)** Molecular subtypes of GBM, **(G)** G-CIMP status in Rembrandt dataset, **(H)** IDH and 1p19q codeletion status in TCGA dataset. ****p<0.0001, ***p<0.001; **p<0.01; *p<0.05; ns, not significant.

REMBRANDT dataset contains normal brain tissues, therefore it was used to validate the expression of different T2Ms in glioma compared to the normal glial tissue. For all subsequent analyses, TCGA along with the REMBRANDT dataset was utilized for clinical correlations. REMBRANDT dataset revealed reduced *MAGEH1* expression in glioma compared to its normal counterpart. Interestingly, analysis of both REMBRANDT and TCGA datasets brought out a steady decrease in *MAGEH1* expression with advancing grades of glioma (**Figures 2A, B**). Furthermore, the analysis of histopathological data revealed a strong correlation of *MAGEH1* downregulation with astrocytoma histology (**Figures 2C, D**). Among TCGA defined molecular subtypes of GBM described by Wang et al. 2017 (38), *MAGEH1* exhibited the highest expression in the proneural subtype while classical and mesenchymal subtypes exhibited its comparable expression (**Figures 2E, F**). Regarding epigenetic alterations, higher *MAGEH1* expression was observed in G-CIMP GBMs compared to non-G-CIMP GBMs in the REMBRANDT dataset (*p*<0.01, **Figure 2G**). In TCGA dataset, *IDH* mutant tumors, which are closely associated with the G-CIMP phenotype, exhibited higher *MAGEH1* expression than non-*IDH* mutant gliomas (*p*<0.001, **Figure 2H**). Furthermore, *IDH* mutant tumors that harbor 1p/19q co-deletion expressed higher levels of *MAGEH1* compared to the non co-deletion tumors with *IDH* mutation (*p*<0.001), which together indicate epigenetic regulation as possible mechanisms of T2Ms expression.

### Alterations in T2M Genes in Glioma

**Figure 3:**
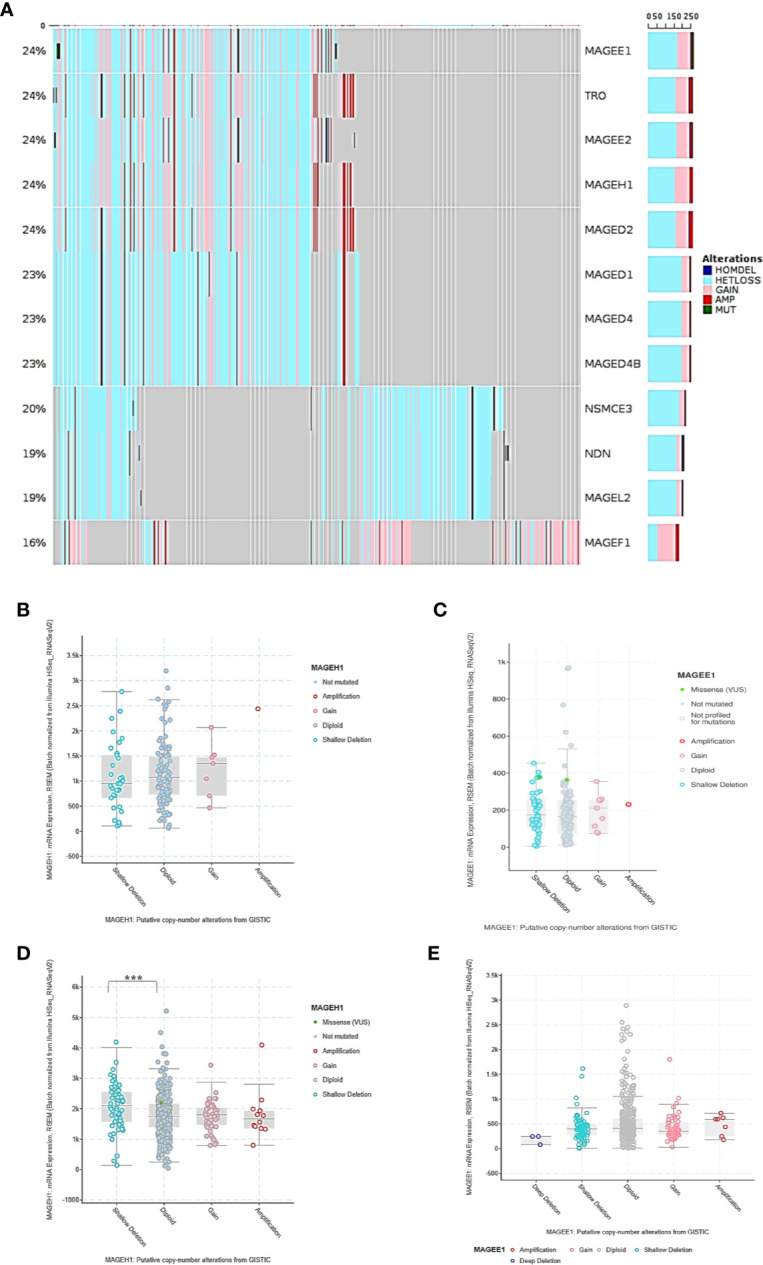
Genetic regulation of T2Ms expression in glioma. **(A)** Mutation and copy number alteration profile of glioma patients from TCGA dataset. **(B, C)** Association of copy number alterations and expression of *MAGEH1* and *MAGEE1* in TCGA-GBM dataset. **(D, E)** Association of copy number alteration and expression of *MAGEH1* and *MAGEE1* in TCGA-LGG dataset. ***p<0.001; results with p>0.05 have not been indicated.

To determine the possible contribution of genetic alterations in altered expression of T2Ms in glioma, copy number variation (CNV), and mutation data of TCGA-LGG (lower grade glioma consisting grade II +III) and TCGA-GBM (glioblastoma) dataset were assessed. Our results showed heterozygous deletion and copy number gain to be common events in T2M genes. However, they rarely undergo mutations, or display amplification and/or deep deletion events (**Figure 3A**). Interestingly, it was also observed that some glioma patients harbor heterozygous deletion of multiple T2Ms. Further, to determine the effect of copy number variation (CNV) on T2Ms expression, we compared their expression in glioma patients based on CNV data and observed no difference in expression of *MAGEH1* (**Figure 3B**) and *MAGEE1* (**Figure 3C**)**** between tumors with diploid and shallow deletion status, and between diploid and copy number gain status for the respective genes. In TCGA-LGG dataset, shallow copy number deletion was indeed associated with higher expression of *MAGEH1* compared to tumors with diploid status for this gene (*p*=0.0009, **Figure 3D**), while no such difference was observed for *MAGEE1* (**Figure 3E**)****.

### NA Methylation of MAGEH1 in Glioma

**Figure 4:**
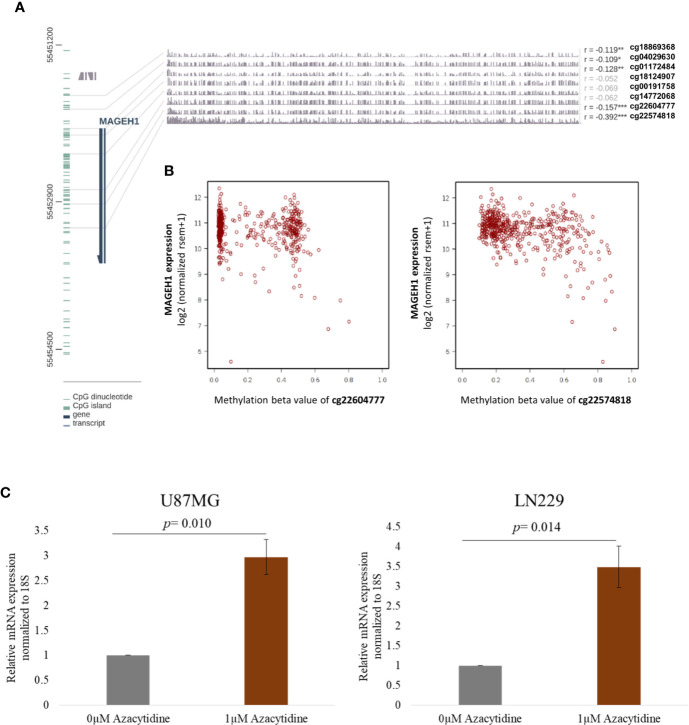
DNA methylation and its correlation with *MAGEH1* expression **(A)** DNA methylation of the *MAGEH1* promoter region in TCGA-LGG dataset **(B)** Correlation of DNA methylation at cg22604777 and cg22574818 with *MAGEH1* expression in TCGA-LGG dataset. **(C)** Effect of 5-Azacytidine treatment on *MAGEH1* expression in GBM cell line U87MG and LN229. ***p<0.001; **p<0.01; *p<0.05. Faded correlation values shows non significant associations.

While DNA methylation is known to regulate *MAGED4* expression in glioma, no information about its role in regulating the expression of other T2M genes is available. We observed that *MAGEH1* expression was associated with *IDH* mutation status and G-CIMP status. Therefore, we utilized DNA methylation data from TCGA-LGG and TCGA-GBM datasets to analyze the association of *MAGEH1* expression with DNA methylation. Correlation analysis was performed between *MAGEH1* expression and its gene methylation. While the *MAGEH1* gene does not contain a CpG island, we observed that in LGG, methylation of CpG sites with probe ids cg18869368, cg04029630, and cg01172484, which have binding sites near TSS of the *MAGEH1* gene were negatively associated with its expression (r= -0.119, r= -0.109 and r= -0.128, respectively, **Figure 4A**). Interestingly, methylation levels of two intragenic CpG sites cg22604777 and cg22574818 exhibited the highest negative correlation with *MAGEH1* expression in LGG (r= -0.157 and r= -0.392, respectively, **Figure 4B**). However, in the GBM dataset, DNA methylation level of only one site (cg01172484) with a binding site near TSS was significantly correlated with gene expression (r= -0.255, **Supplementary Figure S4A**) while comparatively higher negative correlations of gene expression with DNA methylation were observed at intragenic CpG sites cg22604777 and cg22574818 (r= -0.314 and r= -0.505, respectively, **Supplementary Figure S4B**). Treatment of GBM cell line U87MG and LN229 with DNA methyltransferase (DNMT) inhibitor 5-Azacytidine enhanced *MAGEH1* mRNA expression thereby confirming the role of epigenetic regulation in *MAGEH1* expression in glioma (**Figure 4C**).

### Association of T2Ms With Glioma Patient Survival

**Figure 5:**
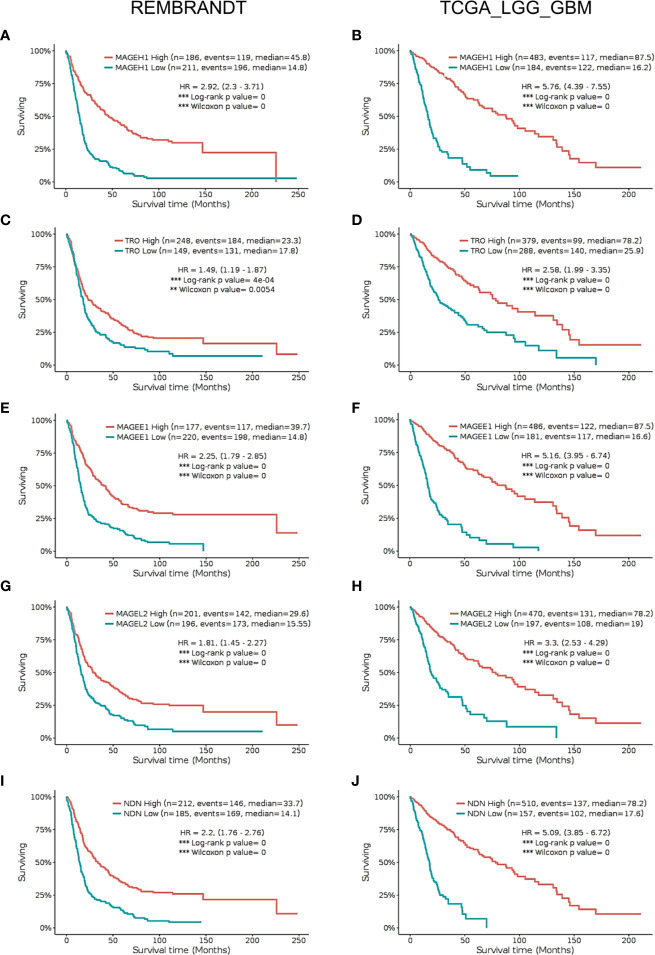
Kaplan Meier survival analysis in REMBRANDT dataset (Left panel) and TCGA dataset (right panel) for selected type 2 MAGE genes including all cases. **(A, B)** *MAGEH1* **(C, D)** *TRO* **(E, F)** *MAGEE1* **(G, H)** *MAGEL2* **(I, J)** *NDN*. Patients were divided into two groups based on maximally selected rank statistics function available in the GlioVis web server (see *Materials and Methods* for detail). The Log-rank p-value has been depicted in each graph. ***p<0.001; **p<0.01; *p<0.05.

Using Kaplan-Meier analysis, we assessed the association of *MAGEH1* expression with overall survival in glioma patients in the REMBRANDT and TCGA-LGG-GBM dataset. Pan-glioma analysis revealed association of *MAGEH1* with better survival in both, REMBRANDT (HR=2.92, 95% CI=2.3–3.71, *p*<0.001, **Figure 5A**) and TCGA dataset (HR=5.76, 95% CI=4.39–7.55, *p*<0.001, **Figure 5B**). We also performed a survival analysis on other T2Ms in REMBRANDT dataset, which revealed that all genes in downregulated T2M subgroup is associated with poor overall survival, such as *TRO* ((HR=1.49, 95%CI=1.19-1.87, *p*<0.001), **Figure 5C**), *MAGEE1* (HR=2.25, 95% CI=1.79–2.85, *p*=0, **Figure 5E**), *MAGEL2* (HR=1.81, 95% CI=1.45–2.27, *p*=0, **Figure 5G**), *NDN* (HR= 2.2, 95% CI=1.76–2.76, *p*=0, **Figure 5I**). Similar association of T2M downregulation with poor OS was observed in TCGA dataset, such as *TRO* (HR=2.58, 95%CI=1.99-3.35, *p*=0, **Figure 5D**), *MAGEE1* (HR=5.16, 95% CI=3.95–6.74, *p*=0 in TCGA dataset, **Figure 5F**), *MAGEL2* (HR=3.3, 95% CI=2.53–4.29, *p*=0 in TCGA dataset, **Figure 5H**) and *NDN* (HR=5.09, 95% CI= 3.85–6.72, *p*=0, **Figure 5J**).

**Figure 6:**
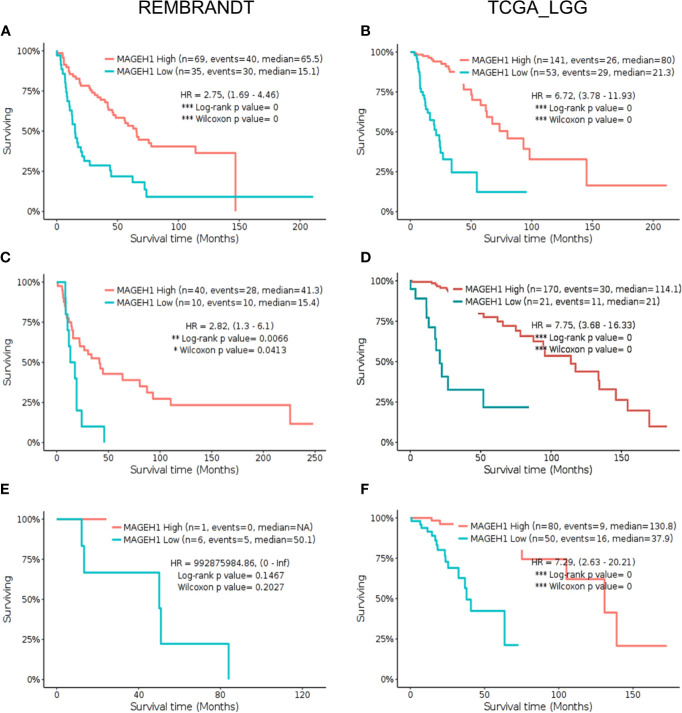
Kaplan Meier survival analysis for *MAGEH1* in different histological subtypes of lower grade glioma from Rembrandt and TCGA dataset **(A, B)** Astrocytoma **(C, D)** oligodendroglioma **(E, F)** mixed glioma. Patients were divided into two groups based on maximally selected rank statistics function available in the GlioVis web server (see *Materials and Methods* for detail). The Log-rank p-value has been depicted in each graph. ***p<0.001; **p<0.01; *p<0.05.

Further, we sought to analyze subgroup specific associations of *MAGEH1* expression with overall survival in different histological subtypes of gliomas. In REMBRANDT dataset, a clear association of *MAGEH1* downregulation with poor OS was observed in astrocytoma (HR=2.75, 95% CI=1.69–4.46, *p*=0, **Figure 6A**) and oligodendroglioma (HR=2.82, 95% CI= 1.3–6.1, *p*<0.01, **Figure 6C**). Since the sample size for mixed glioma (oligoastrocytoma) in the REMBRANDT dataset was small (n=7), no conclusion on the association of *MAGEH1* expression with survival could be drawn (**Figure 6E**). However, in TCGA dataset also, reduced *MAGEH1* expression was associated with poor OS in astrocytoma (HR=6.72, 95% CI=3.78–11.93, *p*=0, **Figure 6B**), oligodendroglioma (HR=7.75, 95% CI = 3.68–16.33, *p*=0, **Figure 6D**) and mixed glioma (HR=7.29, 95% CI=2.63–20.21, *p*=0, **Figure 6F**).

**Figure 7:**
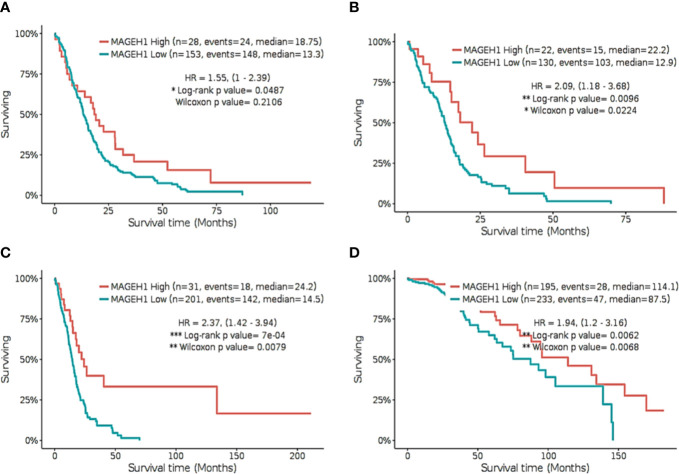
Kaplan Meier survival analysis for *MAGEH1* in Rembrandt and TCGA dataset **(A)** GBM tissues from REMBRANDT **(B)** GBM tissues from TCGA **(C)** IDH wild type from TCGA dataset **(D)** IDH mutant type from TCGA dataset. Patients were divided into two groups based on maximally selected rank statistics function available in the GlioVis web server (see *Materials and Methods* for detail). The Log-rank p-value has been depicted in each graph. ***p<0.001; **p<0.01; *p<0.05.

Further, we separately analyzed the prognostic significance of *MAGEH1* in GBM using REMBRANDT and TCGA datasets. This analysis also brought out the association of reduced *MAGEH1* expression with poor OS in both REMBRANDT (HR=1.55, 95% CI=1–2.39, *p*<0.05, **Figure 7A**), and TCGA (HR=2.09, 95% CI=1.18–3.68, *p*<0.01, **Figure 7B**) dataset. Since *IDH* mutation are associated with poor patient survival in glioblastoma, we separately assessed the prognostic significance of *MAGEH1* in patients harboring these IDH genotypes, Interestingly, reduced *MAGEH1* expression exhibited a strong association with poor OS in glioma patients harboring either wild type (HR=2.37, 95% CI=1.42–3.94, *p*<0.001, **Figure 7C**) or mutant (HR= 1.94, 95% CI=1.2–3.16, *p*<0.01, **Figure 7D**) genotypes.

Additionally, we performed univariate and multivariate Cox regression analysis for prognostic significance of relevant clinicopathological features along with *MAGED1* and *MAGEH1* expression in TCGA-LGG (a combined group of grade II and grade III) and TCGA-GBM datasets, separately. Overall survival (OS), disease specific survival (DSS), disease free interval (DFI) and progression-free interval (PFI) were taken as outcome measures. In the univariate analysis for LGG, we used *MAGEH1* expression, *MAGED1* expression, age, gender, tumor histology, WHO grade, *IDH* mutation status, 1p/19q codeletion status, and *MGMT* promoter methylation status to analyze the association of these variables to patient outcome. Among these, age, tumor grade and astrocytoma histology were associated with poor OS, DSS and PFI, while *IDH* mutation status, 1p/19q codeletion status, and *MGMT* promoter methylation status were associated with better OS, DSS, and PFI (**Table 1**). Additionally, *IDH* mutation status was also associated with DFI. Interestingly, *MAGEH1* expression, but not *MAGED1* expression was associated with better OS, DSS, and PFI in LGG. Furthermore, multivariate analysis revealed that higher *MAGEH1* expression is also an independent prognostic indicator of the better OS, DSS, and PFI in LGG (**Table 2**). On the other hand, multivariate analysis using LGG dataset for *MAGED1* suggested no association of *MAGED1* with patient survival (**Table 3**).

**Table 1:** Univariate survival analysis in TCGA-LGG dataset.

	OS			DSS			DFI			PFI		
	HR	95%CI	*p* value	HR	95%CI	*p* value	HR	95%CI	*p* value	HR	95%CI	*p* value
*MAGEH1* expression	0.342	0.233–0.503	0.000*	0.304	0.200–0.461	0.000*	0.448	0.183–1.098	0.079	0.439	0.327–0.590	0.000*
*MAGED1* expression	0.951	0.669–1.353	0.784	1.001	0.690–1.450	0.994	1.195	0.494–2.892	0.692	0.967	0.727–1.285	0.818
Histology												
OA vs. O (Ref.)	1.080	0.656–1.776	0.761	1.022	0.597–1.752	0.934	0.548	0.159–1.879	0.339	0.832	0.556–1.244	0.372
A vs. O (Ref.)	1.782	1.192–2.663	0.005*	1.902	1.248–2.899	0.003*	1.009	0.374–2.716	0.986	1.605	1.165–2.212	0.004*
Grade												
III vs. II (Ref.)	3.093	2.033–4.705	0.000*	3.187	2.039–4.981	0.000*	0.692	0.280–1.707	0.424	1.554	1.143–2.112	0.005*
*IDH* status												
*IDH* MUT vs. WT (Ref.)	0.153	0.105–0.222	0.000*	0.134	0.091–0.198	0.000*	0.223	0.074–0.673	0.008*	0.173	0.125–0.238	0.000*
1p/19q Co-deletion status												
Present vs. Absent (Ref.)	0.393	0.245–0.628	0.000*	0.368	0.222–0.610	0.000*	0.496	0.165–1.485	0.21	0.437	0.307–0.622	0.000*
*MGMT* promoter methylation status												
Present vs. Absent (Ref.)	0.388	0.265–0.569	0.000*	0.349	0.235–0.518	0.000*	0.817	0.239–2.790	0.747	0.423	0.307–0.582	0.000*
Age (increasing years)	1.058	1.042–1.075	0.000*	1.059	1.042–1.076	0.000*	1.028	0.992–1.066	0.122	1.028	1.016–1.040	0.000*
Gender												
Female vs. Male (Ref.)	0.912	0.619–1.342	0.641	0.899	0.598–1.353	0.613	1.190	0.483–2.934	0.705	1.164	0.858–1.577	0.327

OS, overall survival; DSS, disease specific survival; DFI, disease free interval; PFI, progression free interval; HR, hazard ratio; CI, confidence interval; O, oligodendroglioma; OA, oligoastrocytoma; A, astrocytoma; WT, wild type; MUT, mutant, MGMT; O-6-methylguanine-DNA methyltransferase. * indicates p < 0.05.

**Table 2:** Multivariate survival analysis for MAHGEH1 in TCGA-LGG dataset.

	OS			DSS			DFI			PFI		
	HR	95%CI	*p* value	HR	95%CI	*p* value	HR	95%CI	*p* value	HR	95%CI	*p* value
*MAGEH1* expression	0.497	0.308 –0.803	0.004*	0.433	0.257 –0.730	0.002*	0.394	0.139–1.117	0.08	0.658	0.457482 –0.948871	0.025*
Histology												
OA vs. O (Ref.)	0.896	0.476–1.685	0.734	0.852	0.430–1.689	0.647	0.232	0.041–1.312	0.099	0.679	0.419–1.100	0.116
A vs. O (Ref.)	0.887	0.503–1.563	0.678	0.938	0.513–1.689	0.835	0.495	0.129–1.887	0.303	0.905	0.585–1.399	0.655
Grade												
III vs. II (Ref.)	2.002	1.265–3.167	0.003*	1.968	1.205–3.216	0.007*	0.397	0.148–1.068	0.068	1.037	0.740–1.453	0.831
*IDH* status												
*IDH* MUT vs. WT (Ref.)	0.285	0.150–0.541	0*	0.255	0.130–0.500	0*	0.184	0.035–0.944	0.043*	0.235	0.142–0.389	0*
1p/19q Co-deletion status												
Present vs. Absent (Ref.)	0.615	0.311–1.217	0.163	0.690	0.328–1.449	0.327	0.321	0.074–1.380	0.127	0.640	0.386–1.062	0.084
*MGMT* promoter methylation status												
Present vs. Absent (Ref.)	0.985	0.561–1.726	0.958	0.939	0.529–1.666	0.83	2.417	0.453–12.886	0.301	1.220	0.779–1.911	0.384
Age (increasing years)	1.052	1.034–1.071	0*	1.050	1.031–1.070	0*	1.028	0.984–1.075	0.208	1.019	1.006–1.032	0.004*
Gender												
Female vs. Male (Ref.)	0.911	0.607–1.367	0.654	0.895	0.583–1.375	0.614	0.779	0.287–2.114	0.625	1.158	0.850–1.578	0.351

OS, overall survival; DSS, disease specific survival; DFI, disease free interval; PFI, progression free interval; HR, hazard ratio; CI, confidence interval; O, oligodendroglioma; OA, oligoastrocytoma; A, astrocytoma; WT, wild type; MUT, mutant, MGMT; O-6-methylguanine-DNA methyltransferase. * indicates p < 0.05.

**Table 3:** Multivariate survival analysis for MAHGED1 in TCGA-LGG dataset.

	OS			DSS			DFI			PFI		
	HR	95%CI	*p* value	HR	95%CI	*p* value	HR	95%CI	*p* value	HR	95%CI	*p* value
*MAGED1* expression	1.165	0.768–1.765	0.471	1.277	0.821–1.986	0.277	1.940	0.662–5.681	0.226	1.083	0.792–1.481	0.616
Histology												
OA vs. O (Ref.)	1.222	0.672–2.220	0.51	1.253	0.657–2.390	0.492	0.331	0.070–1.565	0.163	0.766	0.479–1.224	0.265
A vs. O (Ref.)	1.143	0.643–2.031	0.647	1.325	0.720–2.436	0.365	0.593	0.170–2.070	0.413	1.018	0.663–1.565	0.932
Grade												
III vs. II (Ref.)	2.022	1.278–3.198	0.003*	1.982	1.213–3.239	0.006*	0.501	0.189–1.326	0.165	1.049	0.748–1.471	0.778
*IDH* status												
*IDH* MUT vs. WT (Ref.)	0.255	0.132–0.490	0*	0.221	0.111–0.439	0*	0.162	0.027–0.950	0.044*	0.221	0.134–0.366	0*
1p/19q Co-deletion status												
Present vs. Absent (Ref.)	0.538	0.280–1.035	0.064	0.594	0.293–1.207	0.15	0.269	0.064–1.122	0.072	0.568	0.349–0.925	0.023*
*MGMT* promoter methylation status												
Present vs. Absent (Ref.)	1.113	0.621–1.992	0.718	1.094	0.602–1.987	0.767	3.810	0.550–26.353	0.175	1.259	0.797–1.991	0.323
Age (increasing years)	1.054	1.036–1.072	0*	1.052	1.033–1.072	0*	1.040	0.992–1.090	0.099	1.021	1.008–1.034	0.001*
Gender												
Female vs. Male (Ref.)	0.847	0.563–1.274	0.426	0.815	0.528–1.256	0.355	1.217	0.434–3.413	0.708	1.132	0.830–1.543	0.431

OS, overall survival; DSS, disease specific survival; DFI, disease free interval; PFI, progression free interval; HR, hazard ratio; CI, confidence interval; O, oligodendroglioma; OA, oligoastrocytoma; A, astrocytoma; WT, wild type; MUT, mutant, MGMT; O-6-methylguanine-DNA methyltransferase. * indicates p < 0.05.

Next, we performed survival analysis for TCGA-GBM data with *MAGEH1* and *MAGED1* expression, along with age, gender, molecular subtypes (38), IDH mutation status, and *MGMT* promoter methylation status (**Table 4**). In univariate analysis, we observed an association of higher *MAGEH1* expression with favorable PFI, while no association of *MAGED1* was observed with the disease outcome. Additionally, multivariate analysis of the association of *MAGEH1* with PFI revealed only marginal significance (*p*=0.07), while no association of *MAGEH1* was observed with OS, DSS, and DFI (**Table 5**). Similar to univariate analysis, multivariate analysis for prognostic significance of *MAGED1* in TCGA-GBM dataset revealed no association of *MAGED1* expression with patient survival (**Table 6**).

**Table 4:** Univariate survival analysis in TCGA-GBM dataset.

	OS			DSS			PFI		
	HR	95%CI	*p* value	HR	95%CI	*p* value	HR	95%CI	*p* value
*MAGEH1* expression	0.928	0.803–1.071	0.309	0.927	0.796–1.080	0.336	0.828	0.710–0.966	0.017*
*MAGED1* expression	1.220	0.789–1.886	0.37	1.235	0.774–1.696	0.374	0.984	0.650–1.490	0.941
Molecular Subtype									
MS vs. CL (Ref)	0.973	0.630–1.502	0.904	1.208	0.628–1.563	0.970	1.006	0.658–1.536	0 0.977
PN vs. CL (Ref)	0.771	0.492–1.208	0.257	0.694	0.426–1.129	0.141	0.677	0.425–1.077	00.100
*IDH* status									
*IDH* MUT vs. WT (Ref.)	0.235	0.094–0.586	0.002*	0.257	0.102–0.644	0.004*	0.314	0.127–0.777	0.012*
*MGMT* promoter methylation status									
Present vs. Absent (Ref.)	0.556	0.357–0.866	0.009*	0.477	0.295–0.772	0.003*	0.542	0.349–0.842	0.006*
Age (increasing years)	1.026	1.011–1.042	0*	1.025	1.009–1.041	0.002*	1.015	1.001–1.029	0.027*
Gender									
Female vs. Male (Ref.)	1.118	0.764–1.635	0.565	1.117	0.744–1.676	0.592	0.917	0.627–1.340	0.657

OS, overall survival; DSS, disease specific survival; PFI, progression free interval; HR, hazard ratio; CI, confidence interval; CL, classical; MS, mesenchymal; PN, proneural; WT, wild type; MUT, mutant, MGMT; O-6-methylguanine-DNA methyltransferase. * indicates p < 0.05.

**Table 5:** Multivariate survival analysis for *MAGEH1* in TCGA-GBM dataset.

	OS			DSS			PFI		
	HR	95%CI	*p* value	HR	95%CI	*p* value	HR	95%CI	*p* value
*MAGEH1* expression	1.064	0.868–1.305	0.546	1.073	0.865–1.332	0.518	0.843	0.695–1.023	0.084
Molecular Subtype									
MS vs. CL (Ref.)	0.953	0.559–1.625	0.862	0.946	0.534–1.677	0.851	0.934	0.562–1.551	0.794
PN vs. CL (Ref.)	0.819	0.438–1.532	0.534	0.689	0.343–1.383	0.296	0.952	0.516–1.758	0.877
*IDH* status									
*IDH* MUT vs. WT (Ref.)	0.455	0.119–1.731	0.248	0.581	0.148–2.280	0.437	0.453	0.106–1.935	0.285
*MGMT* promoter methylation status									
Present vs. Absent (Ref.)	0.592	0.361–0.971	0.038*	0.501	0.291–0.862	0.013	0.589	0.344–1.007	0.053
Age (increasing years)	1.030	1.008–1.052	0.006*	1.030	1.007–1.054	0.009*	1.011	0.990–1.032	0.300
Gender									
Female vs. Male (Ref.)	1.021	0.626–1.664	0.932	1.054	0.618–1.799	0.845	1.033	0.617–1.730	0.900

OS, overall survival; DSS, disease specific survival; PFI, progression free interval; HR, hazard ratio; CI, confidence interval; CL, classical; MS, mesenchymal; PN, proneural; N, neural; WT, wild type; MUT, mutant, MGMT; O-6-methylguanine-DNA methyltransferase. * indicates p < 0.05.

**Table 6:** Multivariate survival analysis for MAGED1 in TCGA-GBM dataset.

	OS			DSS			PFI		
	HR	95%CI	*p* value	HR	95%CI	*p* value	HR	95%CI	*p* value
*MAGED1* expression	1.319	0.804–2.163	0.272	1.372	0.806–2.334	0.243	0.978	0.603–1.585	0.929
Molecular Subtype									
MS vs. CL (Ref.)	1.085	0.626–1.879	0.771	1.108	0.612–2.004	0.734	0.912	0.540–1.540	0.732
PN vs. CL (Ref.)	0.899	0.513–1.574	0.710	0.770	0.411–1.443	0.416	0.805	0.451–1.437	0.465
*IDH* status									
*IDH* MUT vs. WT (Ref.)	0.465	0.123–1.759	0.260	0.588	0.151–2.293	0.445	0.413	0.096–1.765	0.233
*MGMT* promoter methylation status									
Present vs. Absent (Ref.)	0.606	0.367–1.000	0.050	0.516	0.298–0.894	0.018	0.605	0.356–1.027	0.063
Age (increasing years)	1.033	1.010–1.055	0.003*	1.033	1.009–1.058	0.005*	1.010	0.988–1.032	0.345
Gender									
Female vs. Male (Ref.)	0.980	0.594–1.615	0.938	1.007	0.583–1.740	0.977	1.047	0.623–1.757	0.862

OS, overall survival; DSS, disease specific survival; PFI, progression free interval; HR, hazard ratio; CI, confidence interval; CL, classical; MS, mesenchymal; PN, proneural; WT, wild type; MUT, mutant, MGMT; O-6-methylguanine-DNA methyltransferase. * indicates p < 0.05.

### MAGEH1 Associated Pathways in Glioma

**Figure 8:**
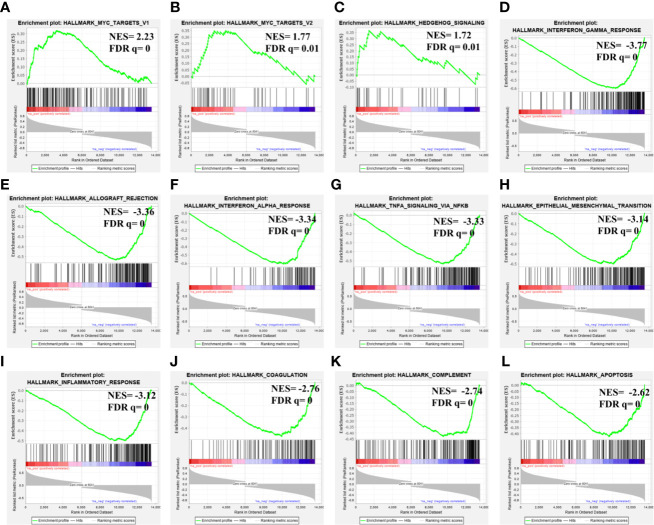
Gene set enrichment analysis for *MAGEH1* associations in lower grade glioma from TCGA dataset. **(A–C)** positively associated pathways, **(D–L)** negatively associated pathways. NES, normalized enrichment score; FDR q, false detection rate corrected p value.

Considering its prognostic significance in LGG, we determined the potential functional associations of *MAGEH1* in LGG by performing gene set enrichment analysis over whole transcriptome correlations of *MAGEH1* expression in TCGA-LGG dataset. Interestingly, positively correlated genes were highly enriched in Myc target genes (**Figures 8A, B**) and Hedgehog signaling (**Figure 8C**). Further, genes negatively correlated to *MAGEH1* expression were enriched in different immune associated pathways (**Figures 8D–G, I–K**) and cancer associated pathways including epithelial to mesenchymal transition (**Figure 8H**) and apoptosis (**Figure 8L**).

### Association of T2M Expression With Immune Response in Glioma

**Figure 9:**
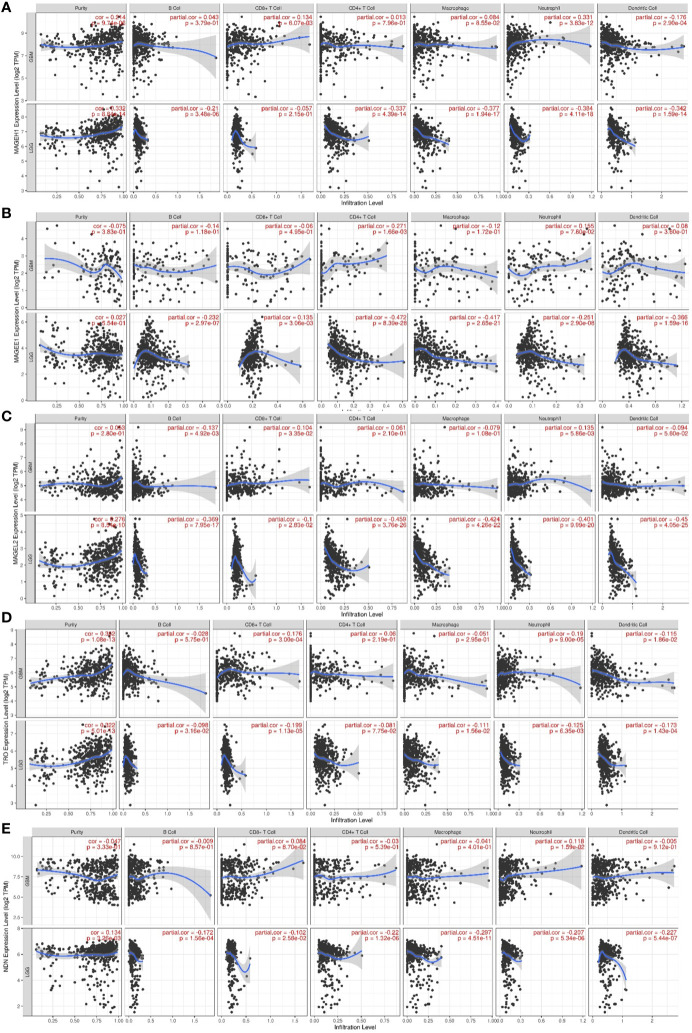
Association of T2M expression with immune infiltration in glioma. TIMER analysis for Type 2 MAGE genes in TCGA-LGG and TCGA-GBM dataset. Analysis outputs for **(A)** *MAGEH1* **(B)** *MAGEE1* **(C)** *MAGEL2* **(D)** *TRO* **(E)** *NDN*.

Pathway analysis revealed that *MAGEH1* correlated genes are involved in tumor immunity. Further, we explored the association of its expression with the level of infiltration of six different immune cell types using RNA deconvolution based TIMER analysis. This analysis in LGG revealed a negative correlation between *MAGEH1* expression and infiltration of multiple immune cells, such as B cells (r= -0.21, p<0.001), CD4 T cells (r=-0.337, p<0.001), macrophages (r=-0.377, p<0.001), neutrophils (r=-0.384, p<0.001), and dendritic cells (r=-0.342, r<0.001) (**Figure 9A**). However in GBM, *MAGEH1* exhibited positive correlation with CD8 T cells (r=0.134, p<0.01) and neutrophils (r=0.331, p<0.001) and a negative correlation with dendritic cells (r=-0.176, p<0.001). Further, other downregulated T2Ms in glioma, including *MAGEE1* (**Figure 9B****)**, *MAGEL2* (**Figure 9C****)**, *TRO* (**Figure 9D****)**, and *NDN* (**Figure 9E****)** also exhibited similar negative correlations with immune cell infiltration in LGG, while in GBM they showed a varying degree of positive correlations with infiltration of different immune cells.

## Discussion

Despite of the recent advances in the understanding of glioma biology, limited improvements have been observed in patient survival (1). The current study was undertaken to analyze the clinical utility of T2Ms expression in glioma. We further focused our analysis to determine the coexpression pattern, underlying regulatory mechanism, and association of T2Ms with tumor immunity. Oncomine analysis provided the benefit of comparing gene expression between tumor and normal brain tissues from multiple datasets, in parallel. This would compensate for the dataset dependent variations and only highly significant observations were considered using a stringent cutoff value of p<0.001. This analysis revealed that some MAGEs including MAGED subfamily are overexpressed, while most others including *MAGEE1*, *MAGEE2*, *MAGEL2*, *MAGEH1*, *NDN*, and *TRO* are downregulated in glioma. The downregulation of these T2Ms suggests their potential tumor suppressor functions in glioma. Downregulation and tumor suppressor functions of Necdin in glioma have been previously reported in detail (19). *MAGEH1* expression has been reported to be downregulated in hepatocellular carcinoma (39) and cholangiocarcinoma (40). Further, *MAGEH1* overexpression inhibits proliferation, cell migration, and invasion in hepatocellular carcinoma (39), induces apoptosis in melanoma cell lines (41), and cell cycle arrest in multiple cancer cell lines (40, 42). In agreement with these studies, we also observed that *MAGEH1* is significantly downregulated with advanced grade of glioma. Further, higher *MAGEH1* was also associated with age, *IDH* mutation, and 1p/19q co-deletion status.

Similar to T1Ms, which are coordinately expressed (23), our correlation analysis revealed that T2Ms are also co-expressed in glioma tissues. Further, we observed highly coordinated expression within two major groups of the MAGE gene family in multiple cancers, including glioma. Interestingly, while MAGED subfamily members showed upregulation in glioma, they also exhibited strong positive correlation among themselves, therefore suggesting common regulation and functions. Theoretically, co-expression of structurally similar MAGE proteins can regulate a large number of oncogenic functions. Nevertheless, functional interaction of two MAGE proteins to perform a single oncogenic function has also been reported (43). Furthermore, both MAGEH1 and Necdin have been shown to interact with p75 neurotrophin receptors, a class of receptors that regulate cell growth and neuronal survival (44).

We hypothesized that the coexpression pattern observed for the downregulated T2Ms is guided by a common regulatory mechanism, including DNA methylation. DNA hypomethylation associated overexpression of *MAGED4* has previously been reported in glioma (18). Therefore, it was to our interest whether the downregulated T2Ms that emerged in the expression analysis are also regulated by DNA methylation. Interestingly, we identified several intragenic CpG sites in *MAGEH1* gene, where DNA methylation was closely associated with its gene expression. Although the IDH mutation in glioma leads to a hypermethylation phenotype and global gene repression, interestingly, *MAGEH1* exhibited higher expression in *IDH* mutant tumors. This is in agreement with previous reports which demonstrated that DNA hypermethylation may also upregulate some genes by promoting transcription accessibility at some genomic regions (45).

The prognostic utility of T2Ms was revealed by survival analysis in TCGA datasets. Interestingly, the association of *MAGEH1* with better survival of patients was separately validated in major histological and molecular subtypes of glioma. Further, this association was independent of *IDH* status and other clinically relevant variables. The association of *MAGED4* overexpression with poor prognosis of glioma patients has been previously reported (46). In agreement to this, our analysis revealed that while *MAGED1*, which is co-expressed with *MAGED4*, was associated with poor prognosis in glioma, it did not emerge as an independent prognostic factor in multivariate analysis. Using a similar analysis of cancer-testis antigens in TCGA datasets, association of Necdin expression with favorable overall survival in glioma has also been reported (20). Our study revealed that NDN expression is downregulated in a coordinated manner with other T2Ms and higher expression of these T2Ms is associated with better patient prognosis in lower grade glioma.

While the current pieces of evidence strongly suggest tumor suppressor functions of some T2Ms, limited information is available for detailed functions of these proteins in glioma. We, therefore, performed pathway analysis for *MAGEH1* co-expressed genes, which revealed strong positive enrichment of Myc and Hedgehog signaling genes. While both of these signaling pathways promote glioma progression (47, 48), their functional association with MAGEH1 remains unclear. Nevertheless, a higher c-Myc expression is indeed associated with better prognosis in glioma patients (49). This might be partially due to less characterized proapoptotic properties of c-Myc, which may sensitize Myc overexpressing glioma cells to DNA damaging agents, such as chemotherapy and radiotherapy (49).

Recent evidence suggests that immune response plays a crucial role in glioma and immunity associated pathways are associated with therapeutic response and clinical outcome. Further, analysis of immunogenomic profiles has led to the identification of several immunomodulatory pathways, such as DNA damage repair (50) and tumor-associated immunomodulatory proteins, such as Galectin-1 and IGFBP2 (51, 52). Pathway analysis revealed that the expression of *MAGEH1* was negatively correlated with several immunity associated pathways along with level of immune infiltration in LGG. Contrary to this, a positive correlation of MAGEH1 with different immune cell population was observed in GBM. These results suggest that T2Ms might be involved in regulating tumor immune infiltration distinctly in LGG compared to GBM. LGG have been shown to exhibit distinct immune composition compared to GBMs (53, 54). In addition to expected intratumor heterogeneity, the microenvironment composition variably influences gene expression profiles (55, 56).

In conclusion, the current study provides intrinsic details of coordinated expression, epigenetic regulation, and prognostic significance of T2Ms in glioma. These findings will provide a strong basis and resource for designing future research on Type 2 MAGE proteins. However, this study is limited by the utilization of gene expression as the primary measure for alterations of T2Ms. Therefore, a protein level study with mechanistic exploration may provide better insights into the role of T2M proteins in glioma biology.

## Data Availability Statement

The datasets used in this study are publicly available. The source datasets are available at the following links: TCGA-LGG and TCGA-GBM dataset, https://portal.gdc.cancer.gov/; REMBRANDT dataset, https://www.ncbi.nlm.nih.gov/geo/query/acc.cgi?acc=GSE108476; Oncomine tool is freely available after registration at https://www.oncomine.org/resource/login.html.

## Ethics Statement

Ethical review and approval was not required for the study on human participants in accordance with the local legislation and institutional requirements. Written informed consent for participation was not required for this study in accordance with the national legislation and the institutional requirements.

## Author Contributions

MA conceptualized the study. SC supervised the study and provided essential infrastructure. MA, SK, and JS and AC performed data curation, interpretation, and statistical analysis. MA and SK wrote the original manuscript. All authors contributed to the article and approved the submitted version.

## Funding

This work is supported by the Wellcome Trust/DBT India Alliance Fellowship (grant number: IA/CPHI/17/1/503333) awarded to AC and project support under young scientist scheme from Department of Health Research, Government of India granted to SK.

## Conflict of Interest

The authors declare that the research was conducted in the absence of any commercial or financial relationships that could be construed as a potential conflict of interest.
