# Supplementary material for: Downregulation of Brain Enriched Type 2 MAGEs Is Associated With Immune Infiltration and Poor Prognosis in Glioma
Source: Front Oncol. 2020 Dec 23;10:573378. doi: 10.3389/fonc.2020.573378 (PMC7787151; doi:10.3389/fonc.2020.573378)
Supplement: Supplementary file 1 [file DataSheet_1.docx]

Supplementary Material

##
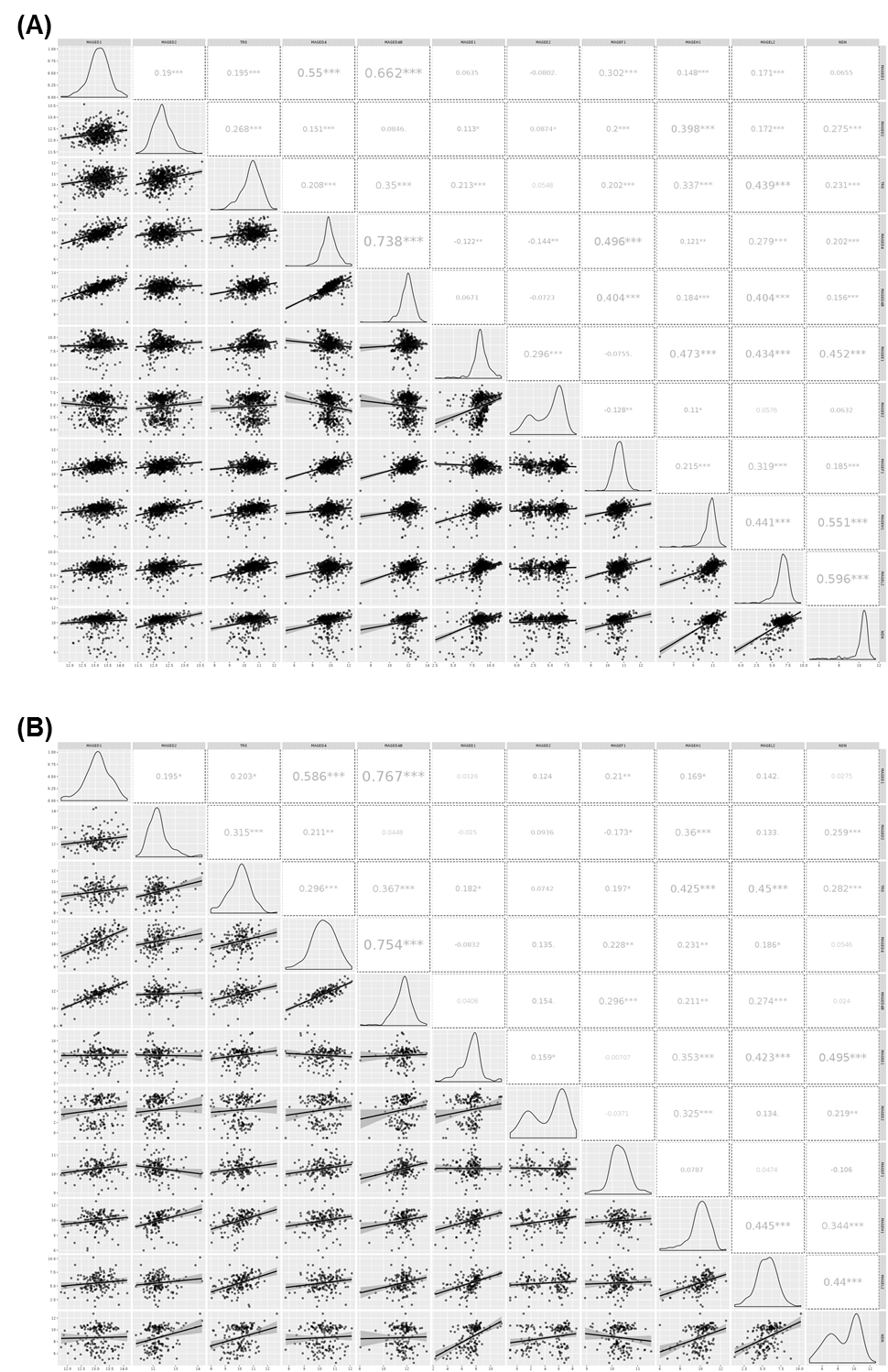
Supplementary Figures

**Supplementary Figure S1.** Correlation matrix of T2Ms in TCGA glioma datasets for (A) low grade gliomas, including grade II and III (B) glioblastoma multiforme. The distribution of variables is shown in left while Pearson correlation coefficients along with the level of significance have been shown on right. ***p<0.001, **p<0.01, *p<0.05.


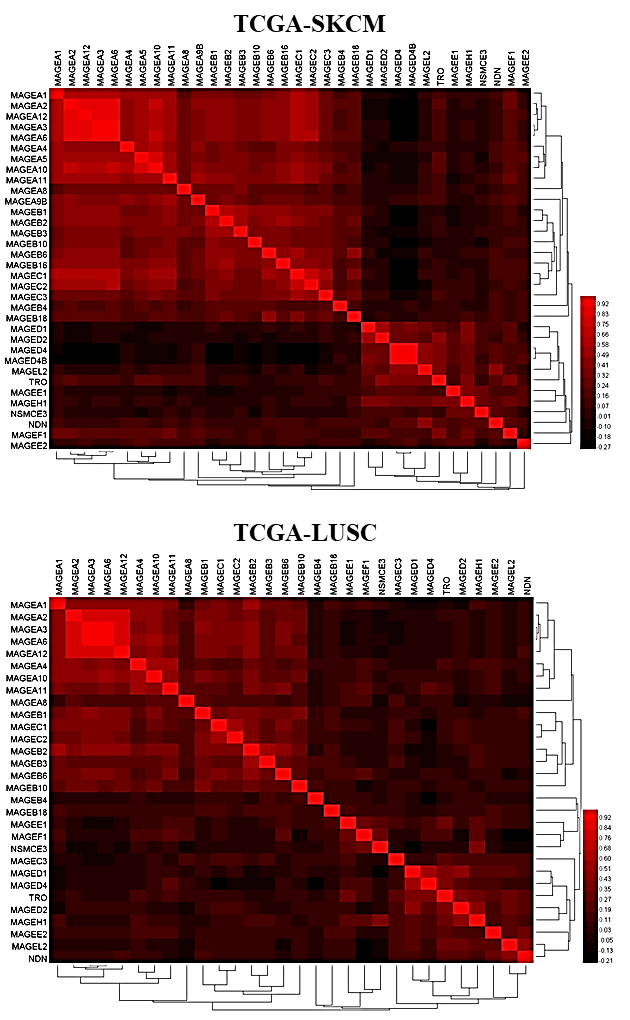

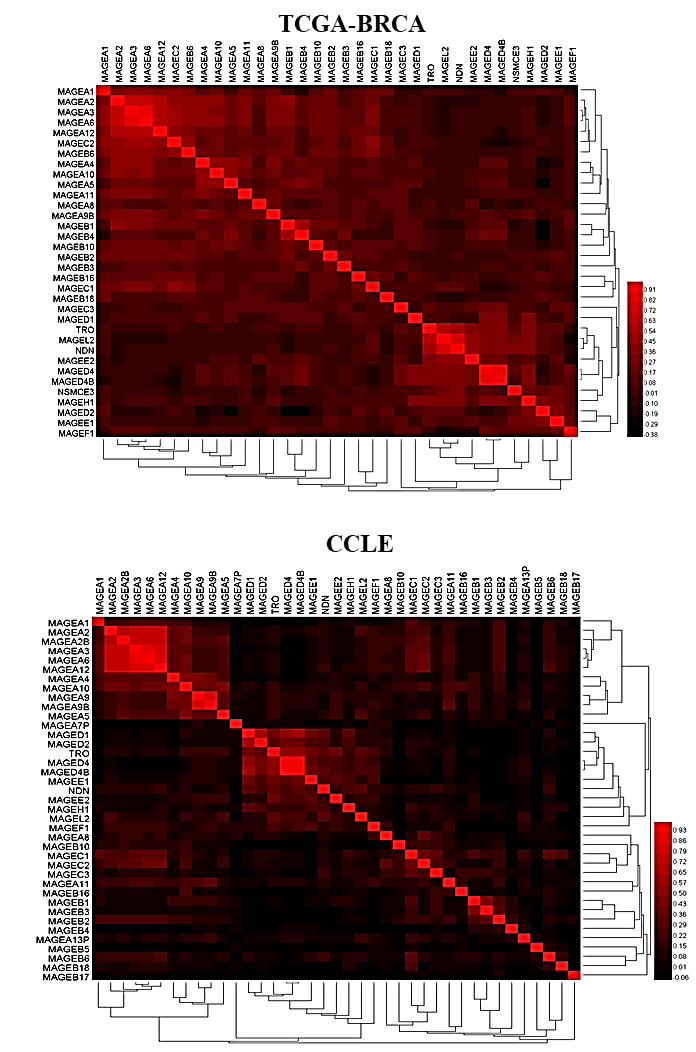


**(A)**

**(B)**


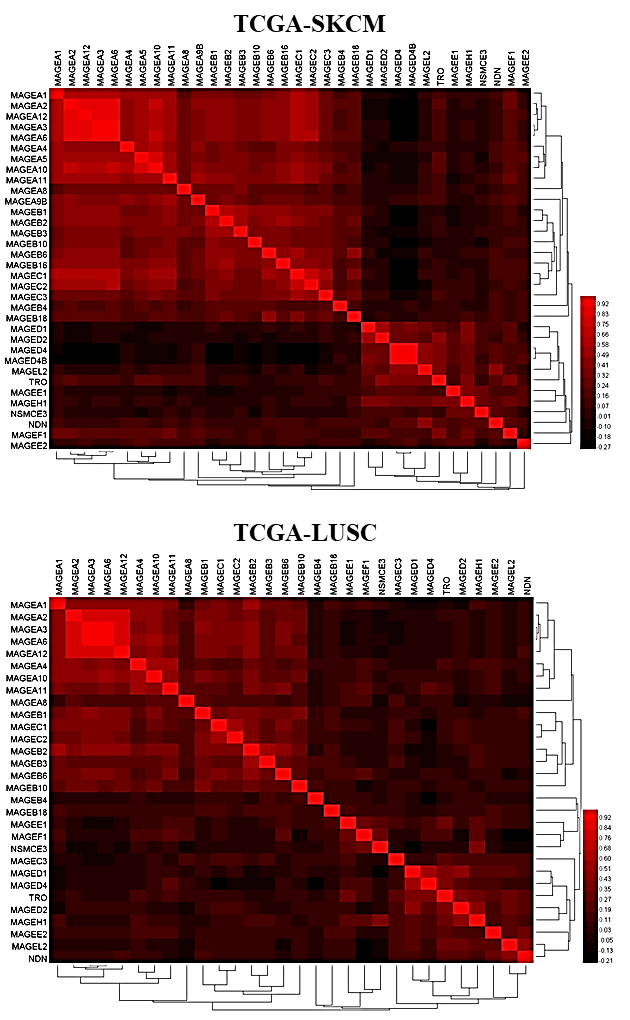

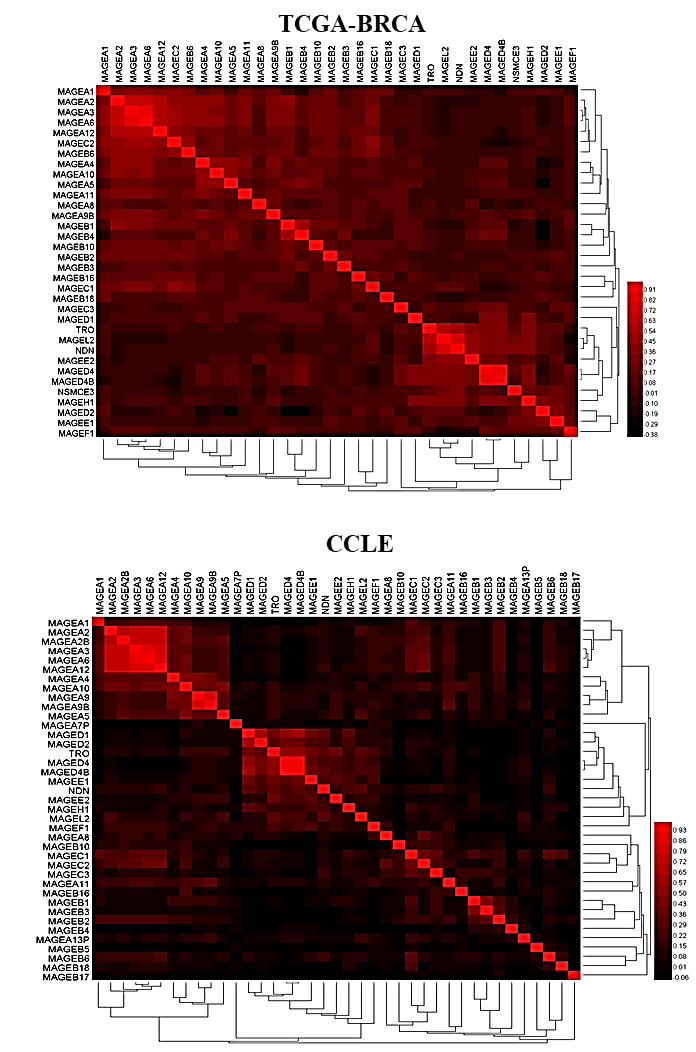


**(D)**

**(C)**

**Supplementary Figure S2.** Coexpression matrix of MAGE family expression in (A) TCGA skin cutaneous melanoma (SKCM) and (B) breast cancer datasets (BRCA), (C) lung squamous cell carcinoma dataset (LUSC) and (D) cell lines established from CCLE.

**(A)**

**(B)**

**(C)**

**(D)**

**(E)**

**Supplementary Figure S3.** Gene expression data of type 2 MAGE genes from different body tissues accessed by The Genotype-Tissue Expression (GTEx) project data (A) *MAGEH1* (B) *MAGEE1* (C) *MAGEL2* (4) *TRO* (E) *NDN***.**

**
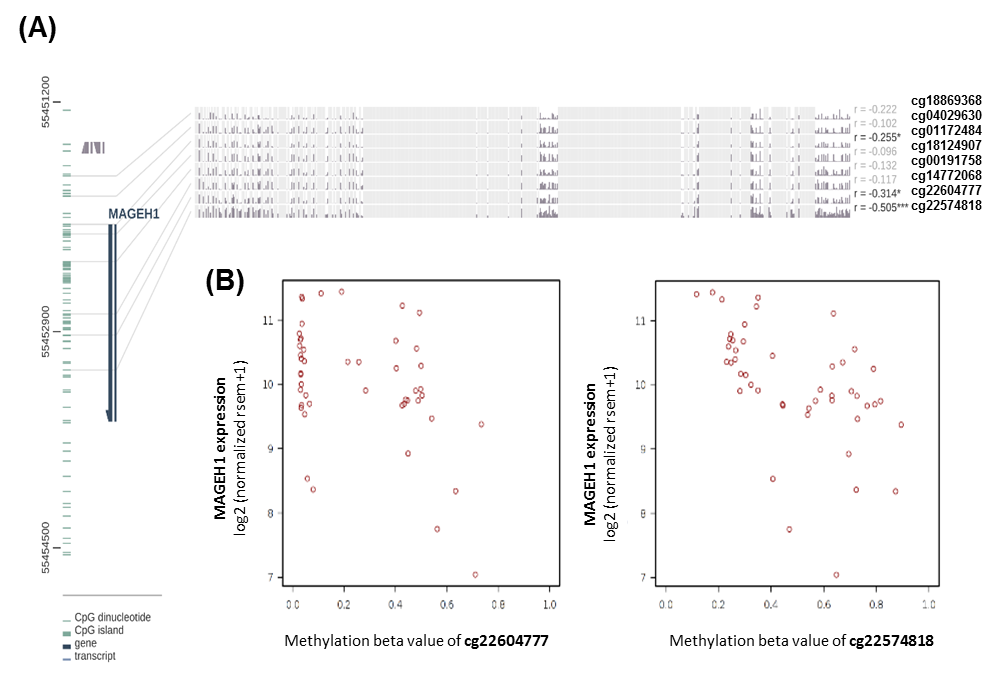
**

**Supplementary Figure S4.** DNA methylation and its correlation with MAGEH1 expression (A) DNA methylation of the MAGEH1 promoter region in TCGA-GBM dataset (B) Correlation of DNA methylation at cg22604777 and cg22574818 with MAGEH1 expression in TCGA-GBM dataset. ***p<0.001, **p<0.01, *p<0.05. Insignificant associations in correlation analysis have been faded.
